# Role of the GacS Sensor Kinase in the Regulation of Volatile Production by Plant Growth-Promoting *Pseudomonas fluorescens* SBW25

**DOI:** 10.3389/fpls.2016.01706

**Published:** 2016-11-18

**Authors:** Xu Cheng, Viviane Cordovez, Desalegn W. Etalo, Menno van der Voort, Jos M. Raaijmakers

**Affiliations:** ^1^Laboratory of Phytopathology, Wageningen UniversityWageningen, Netherlands; ^2^Department of Microbial Ecology, Netherlands Institute of EcologyWageningen, Netherlands; ^3^Institute of Biology Leiden, Leiden UniversityLeiden, Netherlands

**Keywords:** two-component regulation, *Pseudomonas*, GC-MS, volatile organic compounds, plant growth promotion, ISR

## Abstract

In plant-associated *Pseudomonas* species, the production of several secondary metabolites and exoenzymes is regulated by the GacS/GacA two-component regulatory system (the Gac-system). Here, we investigated if a mutation in the GacS sensor kinase affects the production of volatile organic compounds (VOCs) in *P. fluorescens* SBW25 (*Pf*.SBW25) and how this impacts on VOCs-mediated growth promotion and induced systemic resistance of *Arabidopsis* and tobacco. A total of 205 VOCs were detected by Gas Chromatography Mass Spectrometry for *Pf.* SBW25 and the *gacS*-mutant grown on two different media for 3 and 6 days. Discriminant function analysis followed by hierarchical clustering revealed 24 VOCs that were significantly different in their abundance between *Pf.*SBW25 and the *gacS*-mutant, which included three acyclic alkenes (3-nonene, 4-undecyne, 1-undecene). These alkenes were significantly reduced by the *gacS* mutation independently of the growth media and of the incubation time. For *Arabidopsis*, both *Pf.*SBW25 and the *gacS*-mutant enhanced, via VOCs, root and shoot biomass, induced systemic resistance against leaf infections by *P. syringae* and rhizosphere acidification to the same extent. For tobacco, however, VOCs-mediated effects on shoot and root growth were significantly different between *Pf.*SBW25 and the *gacS*-mutant. While *Pf.*SBW25 inhibited tobacco root growth, the *gacS*-mutant enhanced root biomass and lateral root formation relative to the non-treated control plants. Collectively these results indicate that the sensor kinase GacS is involved in the regulation of VOCs production in *Pf.*SBW25, affecting plant growth in a plant species-dependent manner.

## Introduction

Microorganisms produce a variety of volatile organic compounds (VOCs), which are defined as low molecular weight compounds with high vapor pressure (Cordovez et al., 2015; Schmidt et al., 2015). Their physical and chemical properties allow dispersal over longer distances than other extracellular microbial metabolites. VOCs are structurally diverse and include many yet unknown compounds. For example, from 12 *Streptomyces* strains isolated from the plant rhizosphere, a total of 381 VOCs were detected with most of them structurally unknown (Cordovez et al., 2015). To date, microbial VOCs have been grouped into hydrocarbons, ketones/alcohols, acids, sulfur compounds, nitrogen-containing compounds and terpenes (Audrain et al., 2015; Schmidt et al., 2015). Bacteria can also release inorganic volatiles such as hydrogen cyanide (HCN), ammonia, and nitrous oxide (Audrain et al., 2015; Schmidt et al., 2015). Based on the structural diversity, several natural functions have been proposed for microbial VOCs. These include a role of VOCs as: (1) infochemicals in inter- and intra-organismal communications (Chernin et al., 2011; Schmidt et al., 2015), (2) antimicrobial agents (Kai et al., 2010; Cordovez et al., 2015), and (3) compounds that promote or inhibit plant growth (Blom et al., 2011; Cordovez et al., 2015). Indeed, several bacterial genera, including *Bacillus, Pseudomonas, Streptomyces, Serratia, Arthrobacter, Collimonas*, and *Stenotrophomonas*, can influence plant growth via VOCs (Ryu et al., 2003; Garbeva et al., 2014; Audrain et al., 2015; Cordovez et al., 2015; Kanchiswamy et al., 2015; Park et al., 2015). The bacterial VOCs acetoin and 2,3-butanediol from *Bacillus* are well-known for their role in plant growth promotion and induction of systemic resistance (ISR) against pathogen infection (Ryu et al., 2003, 2004). Over the past years, several other bacterial VOCs, including indole, 1-hexanol, pentadecane, 13-tetradecadien-1-ol, 2-butanone, and 2-methyl-n-1-tridecene, have been implicated in plant growth promotion (Blom et al., 2011; Park et al., 2015).

Bacterial VOCs are synthesized via diverse pathways including aerobic, heterotrophic carbon metabolism, fermentation, amino-acid catabolism, terpenoid biosynthesis, fatty acid degradation, or sulphur reduction (Penuelas et al., 2014). Because the production of certain VOCs appears to be dependent on cell density, quorum sensing (QS) has been suggested as a possible regulatory system of bacterial VOCs production (Audrain et al., 2015). For example, 2-amino-acetophenone (2-AA) produced by *P. aeruginosa* is controlled by the **m**ultiple **v**irulence **f**actor **r**egulator (MvfR), a known QS system (Kesarwani et al., 2011; Que et al., 2013). In contrast, no significant effects on VOCs production were found for a QS-mutant of *Burkholderia ambifaria* LMG19182 (Groenhagen et al., 2013). In *Pseudomonas*, the production of various secondary metabolites and exoenzymes is under the regulation of the GacS/GacA two-component regulatory system (referred to here as the Gac-system). The Gac-system consists of the membrane-bound sensor kinase GacS and the cytoplasmic transcriptional response regulator GacA. Mutations (spontaneous or site-directed) in the *gacS* or *gacA* genes generally abolish secondary metabolite production (Zuber et al., 2003). In *P. protegens* Pf-5, *P. chlororaphis* 30-84, and *P. fluorescens* strains SBW25 (*Pf.*SBW25) and F113, mutations in the *gacA* or *gacS* genes have significant effects on iron homeostasis and expression of genes involved in virulence, biofilm formation, motility, stress responses, and survival (Chancey et al., 1999; Martínez-Granero et al., 2005, 2006; Blom et al., 2011; Cheng et al., 2013; Wang et al., 2013). Production of the volatile HCN is regulated by the Gac-system in *P. chlororaphis* 30-84, *P. fluorescens* F113, and *P. protegens* strains CHA0 and Pf-5 (Chancey et al., 1999; Aarons et al., 2000; Duffy and Defago, 2000; Hassan et al., 2010) and also the production of 2R, 3R-Butanediol by *P. chlororaphis* O6 was shown to be Gac-dependent (Han et al., 2006). To date, however, the role of the GacS sensor kinase in the overall regulation of VOCs produced by plant growth-promoting rhizobacteria is not known. In this study, we analysed the VOC profiles of wild-type *Pf.*SBW25 and its *gacS* mutant grown on different media and after different incubation periods. We subsequently determined if and how a mutation in the *gacS* gene affects VOCs-mediated growth promotion and rhizosphere acidification of *Arabidopsis* and tobacco, and ISR in *Arabidopsis*.

## Materials and Methods

### Bacterial Strains, Media, and Culture Conditions

*Pseudomonas fluorescens* SBW25 (*Pf.*SBW25) and its *gacS-*mutant (referred to here as the Gac-mutant) (Cheng et al., 2013, 2015) were pre-cultured in liquid King’s B (KB) broth supplemented with rifampicin (50 μg/ml) and with rifampicin (50 μg/ml) and kanamycin (100 μg/ml), respectively, at 25°C for 24 h. The pathogen *Pseudomonas syringae* pv. *tomato* DC3000 (*Pst*) was cultured in KB broth supplemented with rifampicin (50 μg/ml) at 25°C for 24 h. Bacterial cells were collected by centrifugation, washed three times with 10 mM MgSO_4_ and resuspended in 10 mM MgSO_4_ to a final density of OD_600_ = 1.0 (∼10^9^ CFU/ml).

### Collection and Analysis of VOCs

For the collection of bacterial VOCs, 100 μL of a cell suspension of *Pf.*SBW25 and the Gac-mutant (OD_600_ = 0.1) were inoculated individually in 90-mm-diameter glass Petri dish containing 20 ml of KB agar or 1/5th strength Potato Dextrose Agar (1/5th PDA, Oxoid) media with three replicates each. Plates containing the agar media only served as controls. To collect the headspace VOCs, the lid of these Petri dishes were designed with an outlet connected to the traps filled with an adsorbent (Tenax). The Tenax traps were pre-conditioned at 260°C with a Helium flow rate for 45 min and cooled afterward to room temperature. Petri dishes connected to the Tenax traps were sealed with parafilm and incubated at 25°C. After 3 and 6 days of incubation, headspace VOCs was analyzed by GC-Q-TOF-MS (Agilent 7890B GC and the Agilent 7200A QTOF, Santa Clara, CA, USA). VOCs were thermally desorbed from the Tenax traps using an automated thermal desorption unit (model UnityTD-100, Markes International Ltd., Llantrisant, UK) at 210°C for 12 min (He flow 50 ml/min) and captured on a cold trap at -10°C. The compounds released were transferred onto the analytical column (30 mm × 0.25 mm ID RXI-5MS, film thickness 0.25 μm – Restek 13424-6850, Bellefonte, PA, USA) with a split ratio of 1:20 (v/v). The temperature program of the GC oven started at 39°C (2-min hold) and rose to 95°C at a rate of 3.5°C min^-1^, to 165°C at 6°C min^-1^, to 250°C at 15°C min^-1^ and finally to 300°C at 40°C min^-1^ (20 min-hold). VOCs were detected by the MS operating at 70 eV in EI mode. Mass scanning was done from 30 to 400 *m/z* with a scan time of 4 scans s^-1^.

Mass signals from GC-MS raw data that were generated using an untargeted metabolomics approach were extracted and aligned by MetAlign software (Lommen and Kools, 2012). MSClust was used to remove signal redundancy per metabolite and to reconstruct compound mass spectra as previously described (Tikunov et al., 2012). VOCs detected for both the media and the bacterial strains [Fold Change (FC) < 2] were removed from the analyses. VOCs were annotated by comparing their mass spectra with those of commercial NIST14 (National Institute of Standards and Technology, USA^1^) and Wiley database 9th edition. The linear retention indices (RI) and the accurate mass of selected VOCs were compared with those in the library. Processed VOCs data were log transformed and auto-scaled using the average as an offset and the standard deviation as scale [raw value-average (offset)/SD (scale)] with GeneMaths XT Version 2.11 (Applied Maths, Belgium)). Log transformed data were subjected to One-Way ANOVA and VOCs that showed a significant difference (*P* < 0.05) at least under one of the conditions analysed were used in the hierarchical cluster analysis (Pearson’s correlation coefficient with UPGMA algorithm) and discriminant analysis. To select VOCs affected by the Gac-mutation, Student’s *t*-Test (for independent samples) was performed between wild-type and mutant for both media and time points following the criteria: *P* < 0.05 (*t*-Test), peak intensity of at least 10^4^ and fold change (FC) > 2.

### VOCs-Mediated Plant Growth Promotion

To determine the role of the GacS in VOCs-mediated growth promotion of *Arabidopsis* and tobacco (*Nicotiana benthamiana*), seedlings were exposed to the VOCs emitted by the wild type *Pf.*SBW25 or the Gac-mutant. *Arabidopsis* seeds (Col-0) were surface sterilized and were sown on square plates (100 × 100 mm) containing 50 ml of half-strength Murashige and Skoog (½MS) agar medium (Murashige and Skoog, 1962) supplemented with 0.5% (w/v) sucrose as previously described (Van De Mortel et al., 2012). Plates were sealed with parafilm and incubated in a climate chamber (21°C/21°C day/night temperature; 250 μmol light m^-2^s^-1^ at plant level during 16 h/d; 70% relative humidity). Dual-dish plates were prepared with a large round Petri dish (145-mm-diameter) containing 100 ml ½ MS medium fixed with a small Petri dish (35-mm-diameter) positioned inside the large Petri plate. *Arabidopsis* or tobacco seedlings were grown in the large Petri plate, whereas in the small Petri plate the bacteria were cultured on KB agar medium. Six days after sowing of surface-sterilized *Arabidopsis* seeds, 10 μl of bacterial suspension (∼10^9^ CFU/ml) was spot-inoculated in the small petri dishes and incubated at 25°C overnight. On the next day, the 7-day-old *Arabidopsis* seedlings were transferred from square Petri dish to half strength MS media in the large Petri plate. Using this experimental set-up, the plants and bacterial cultures were physically separated in the dual-dish plate and bacteria-plant interactions were only possible via VOCs. Plates were sealed with parafilm and then incubated in the climate chamber (21°C/21°C day/night temperature; 250 μmol light m^-2^s^-1^ at plant level during 16 h/d; 70% relative humidity). After 11 days of incubation, plant fresh, and dry weights were determined. Differences in shoot and root biomass were analysed statistically by one-way ANOVA, Tukey, *P < 0.05*). The experiment was performed three times, each time with at least five replicates per treatment. Experiments with tobacco seedlings were performed twice as described above for *Arabidopsis*.

### VOCs-Mediated Induced Systemic Resistance

For the induced resistance (ISR) assay, leaves of 14-day-old *Arabidopsis* seedlings, exposed (or not) to VOCs from wild type *Pf.*SBW25 or the Gac-mutant, were inoculated in the centre of the leaf rosette with 2 μl cell suspension (∼10^9^ CFU/ml) of the pathogen *Pst*. Five to 7 days after inoculation, disease incidence was assessed by determining the percentage of diseased leaves per plant. Leaves were scored as diseased when they exhibited necrotic or water soaked lesions surrounded by chlorotic or necrotic leaf tissue. Disease incidence was calculated for each plant with at least 20 plants per treatment. The experiment was performed at least twice. Statistically significant differences were determined by ANOVA (*P* < 0.05).

### VOCs-Induced Rhizosphere Acidification

Next to the plant growth promotion and ISR assays, pH changes in the rhizosphere of *Arabidopsis* and tobacco seedlings were monitored by supplementing bromocresol green (0.02% w/v) into the ½ MS medium on which the seedlings were grown. Bromocresol green acts as a pH indicator with a yellow color at pH 3.8 and a blue color at pH 5.4. The pH changes were monitored and captured during 11 days of co-cultivation of *Arabidopsis* and tobacco seedlings with the bacterial strains.

## Results

### Gac-Regulation of VOCs Production by *Pf.*SBW25

**FIGURE 1:**
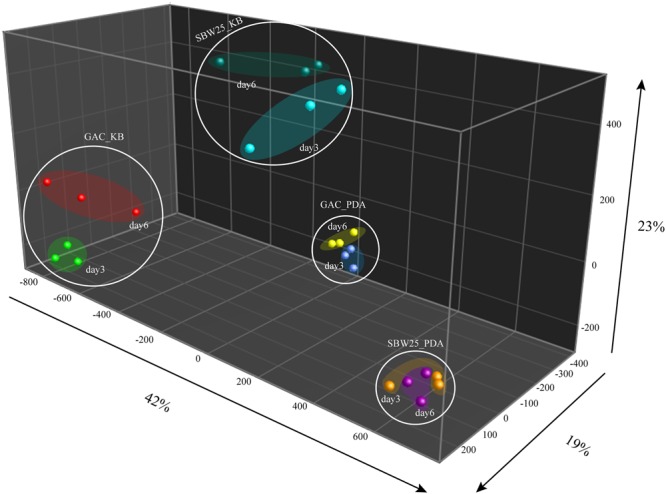
**Discriminant function analysis based on 79 volatile organic compounds (VOCs) that were significantly different in their abundance at least under one of the conditions analyzed (ANOVA, *P < 0.05*).** The first component explained 42% of the total variation that is primarily associated to variation in the abundance of VOCs when *Pf.*SBW25 and the Gac-mutant are grown in 1/5th PDA and KB media. The second component explained 23% of the total variation and particularly related to the effect of the mutation on VOCs when *Pf.*SBW25 and the Gac-mutant are grown on KB medium. The third component explained 19% of the total variation and is related to the effect of the Gac-mutation on the VOCs when *Pf.*SBW25 and the Gac-mutant are grown on 1/5th PDA.

**FIGURE 2:**
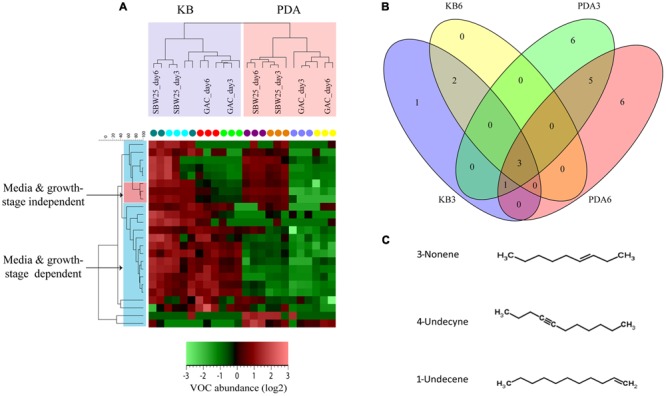
**VOCs profiling of *Pf*.SBW25 and the *gacS*-mutant (Gac-mutant). (A)** Hierarchical cluster analysis (HCA) based on 24 VOCs that were significantly different (*P* < 0.05 and fold change > 2) for the *Pf.*SBW25 and the Gac-mutant grown on 1/5th PDA and KB media after 3 and 6 days of inoculation. Columns represent the different isolates (in triplicates), whereas rows represent the VOCs (green: low abundance, red: high abundance). **(B)** Venn diagram showing the unique and shared VOCs detected for *Pf*.SBW25 and the Gac-mutant grown on 1/5th PDA and KB media after 3 (D3) and 6 (D6) days of inoculation. Yellow and blue cycles represent VOCs detected for strains grown on KB at D3 and D6, respectively; Green and red cycles represent VOCs detected for strains grown on 1/5th PDA at D3 and D6, respectively. **(C)** Group of alkenes significantly affected by the Gac-mutation of *Pf.*SBW25 grown on KB and 1/5th PDA media at D3 and D6 after inoculation.

Volatile organic compounds produced by wild type *Pf.*SBW25 and its Gac-mutant grown on KB or 1/5th PDA agar media were collected at different time points (3 or 6 days of incubation). A total of 205 putative VOCs were detected by GC-MS in the headspace of *Pf.*SBW25 and its Gac-mutant, of which 79 VOCs differed significantly in abundance (One-way ANOVA, *P* < 0.05) in at least one of the growth conditions (**Figure 1**). Discriminant function analysis of these 79 VOCs resulted in a model with 3 principal components, which explained 84% of the total variation. The first component explained 42% of the total variation and is related to the effect of different cultivation media on the VOCs produced by *Pf*.SBW25 and the Gac-mutant. VOCs detected for both *Pf.*SBW25 and the Gac-mutant were clearly separated by the type of cultivation medium (**Figure 1**). The second component explained 23% of the total variation and is related to the effect of the Gac-mutation on VOCs when grown on KB medium. The third component explained 19% of the total variation and is related to the effect of the Gac-mutation on VOCs when the strains are grown on 1/5th PDA (**Figure 1**). A total of 24 VOCs were significantly (*P* < 0.05, FC > 2) affected by the Gac-mutation at least under one of the growth conditions (**Table 1**). The VOC profiles of *Pf.*SBW25 and its Gac-mutant are shown in the Hierarchical Cluster Analysis (HCA; **Figure 2A**). For the majority of these metabolites, the effect of the Gac-mutation on VOCs emission was dependent on the cultivation medium (**Figures 2A,B**).

**Table 1:** List of 24 volatile organic compound (VOCs) significantly affected by the Gac-mutation of *Pf.*SBW25 under different growth conditions and incubation periods.

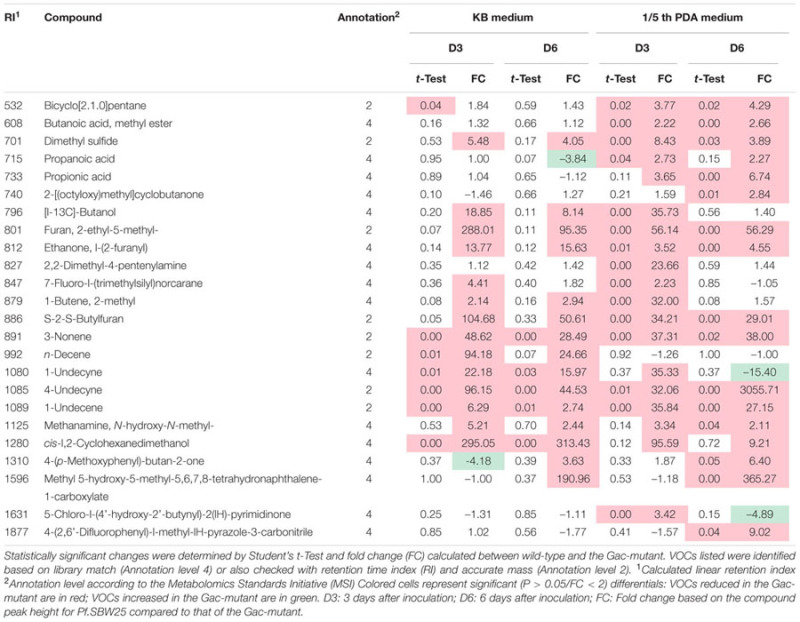

On KB medium, the emission of 3 VOCs belonging to the class of acyclic alkenes was significantly reduced in the Gac-mutant (**Table 1**). These VOCs are 3-nonene (RI: 891), 1-undecene (RI: 1089), and 4-undecyne (RI: 1085). The VOC *n*-decene (RI: 992) was only detected at 3 days of incubation (**Table 1**; **Figure 2C**). On 1/5th PDA, the emission of 7 VOCs was significantly reduced in the Gac-mutant, independent of the incubation time (**Table 1**). These VOCs are bicyclo[2.1.0]pentane (RI: 532), dimethyl sulfide (RI: 701), S-2-S-butylfuran (RI: 886) and the three alkenes described above for KB medium.

### VOCs-Mediated Effects on Plant Growth and ISR

**FIGURE 3:**
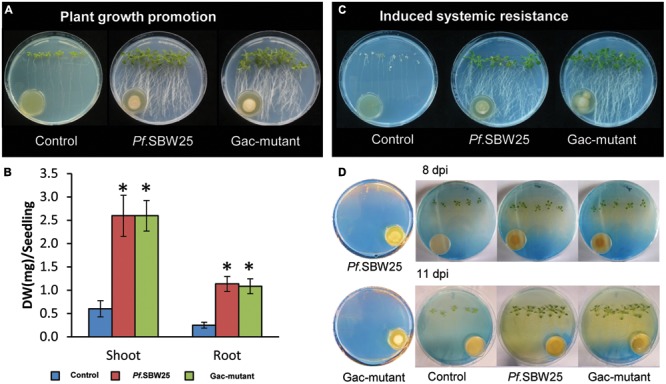
**Growth promotion of *Arabidopsis* after 11 days of exposure to VOCs of *Pf.*SBW25 or the Gac-mutant **(A)**.** Quantitative analysis of the dry weight (DW) of *Arabidopsis* after 11 days of exposure to VOCs of *Pf.*SBW25 or the Gac-mutant **(B)**. Bars represent standard deviation of the mean of 4 independent biological replicates (with 8–10 seedlings per replicate). Asterisks indicate a statistical difference as compared to controls (exposed to medium only) using Student’s *t*-Test (*p* < 0.05, *n* = 4). Induced systemic resistance of *Arabidopsis* against the leaf pathogen *Pseudomonas syringae* pv. tomato *(Pst)* **(C)**. Rhizosphere acidification of *Arabidopsis* after 8 or 11 days of exposure to VOCs of *Pf.*SBW25 or the Gac-mutant **(D)**. Orange colour of the medium reflects pH decline.

Since the Gac-mutant established much lower cell densities than wild-type *Pf.*SBW25 on 1/5th PDA medium but not on KB medium, the plant growth-promotion and ISR assays were performed with the bacterial strains grown on KB only. When *Arabidopsis* seedlings were exposed to VOCs from *Pf.*SBW25 or from the Gac-mutant, plant growth, and root architecture were altered: the primary roots were longer and more lateral roots were formed by the VOCs-exposed plants than by non-exposed (medium only) control plants (**Figure 3A**). Both shoot and root biomass of *Arabidopsis* seedlings, exposed to VOCs from either *Pf.*SBW25, or the Gac-mutant, increased approximately fourfold as compared to control plants (**Figure 3B**). Leaves of *Arabidopsis* plants exposed to VOCs from *Pf*.SBW25 or the Gac-mutant were also visually greener than those of control plants (**Figure 3A**). To test for VOCs-mediated ISR, *Arabidopsis* leaves were inoculated with a suspension of the pathogen *Pst*. Results showed that *Pst* disease incidence was significantly reduced in plant seedlings exposed to VOCs from either *Pf.*SBW25 or from the Gac-mutant (**Figure 3C**). Collectively, these results show that a mutation in the GacS sensor kinase of *Pf.*SBW*25* did not alter VOCs-mediated growth promotion of *Arabidopsis* seedlings nor ISR against *Pst*.

### VOCs-Mediated Acidification of the Rhizosphere

When bromocresol green was added to the plant growth medium as a pH indicator, we observed more rhizosphere acidification (yellowish discoloration) in the VOCs-exposed *Arabidopsis* plants than the control plants (**Figure 3D**). This difference may, for a large part, be explained by the substantial increase in root biomass of the seedlings exposed to VOCs from *Pf.*SBW25 or the Gac-mutant. However, acidification in the *Pf.*SBW25-exposed seedlings was observed not only around the roots but across the entire plate, whereas for the Gac-mutant-exposed plants acidification was located mostly in the area surrounding the roots (**Figure 3D**). In the absence of the *Arabidopsis* seedlings, *Pf.*SBW25 and the Gac-mutant did not cause acidification of the medium. These results suggest that *Pf.*SBW25 and the Gac-mutant induce a quantitatively different VOCs-mediated rhizosphere acidification in *Arabidopsis*.

### VOCs-Mediated Effects on Tobacco

**FIGURE 4:**
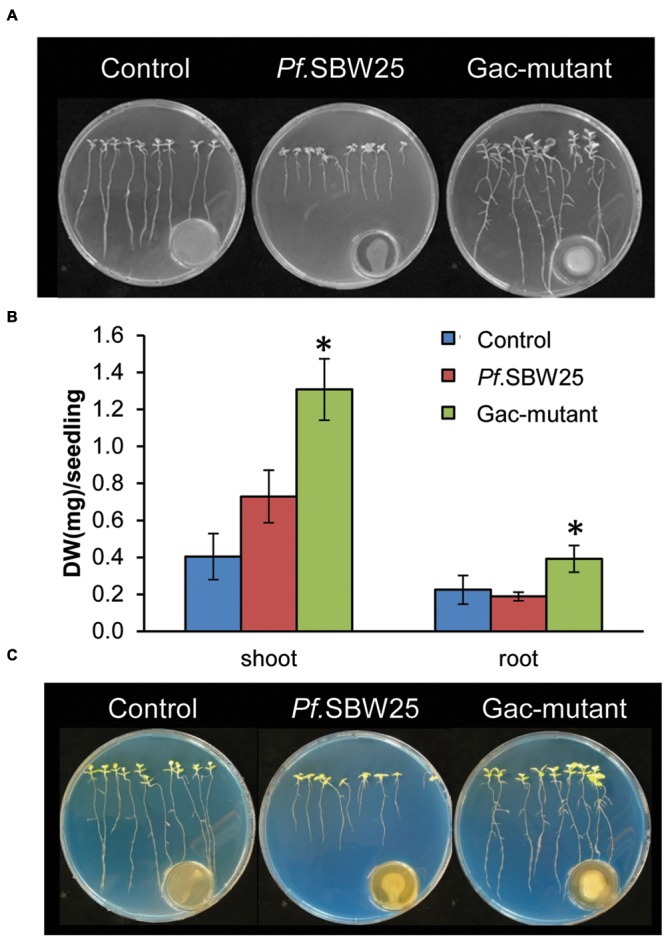
**Growth promotion of tobacco (*Nicotiana benthamiana*) after 11 days of exposure to VOCs of *Pf.*SBW25 or the Gac-mutant **(A)**.** Quantitative analysis of the dry weight (DW) of tobacco after 11 days of exposure to VOCs of *Pf.*SBW25 or the Gac-mutant **(B)**. Bars represent standard deviation of the mean of 4 independent biological replicates (with 8–10 seedlings per replicate). Asterisks indicate a statistical difference as compared to controls (exposed to medium only) using Student’s *t*-Test (*p* < 0.05, *n* = 4). Rhizosphere acidification of tobacco after 11 days of exposure to VOCs of *Pf.*SBW25 or the Gac-mutant **(C)**. Blue colour of the medium reflects no pH decline.

To investigate if the observed VOCs-mediated phenotypic changes by *Pf.*SBW25 are typical for *Arabidopsis* or also appear in other plant species, we conducted similar assays with tobacco (*N. benthamiana*). When tobacco seedlings were exposed to VOCs of *Pf.*SBW25 or the Gac-mutant, the root architecture was differentially affected. When exposed to VOCs from *Pf.*SBW25, growth of the primary roots was inhibited (**Figure 4A**). This apparent visual difference, however, was not supported by quantitative measurements of the root biomass (**Figure 4B**). In contrast, VOCs from the Gac-mutant promoted root growth and induced more lateral roots relative to the *Pf.*SBW*25* and control treatments (**Figure 4A**). Biomass quantifications of both shoot and root supported this latter observation (**Figure 4B**). For tobacco, no VOCs-mediated rhizosphere acidification was observed (**Figure 4C**). Collectively, these results indicate that VOCs-mediated plant growth promotion and rhizosphere acidification by *Pf.*SBW25 is plant species specific.

## Discussion

Microbial VOCs act as infochemicals in microbe-microbe interactions influencing inter- and intra-organismal communications (Schmidt et al., 2015). They can also have antagonistic activities by inhibiting bacterial and fungal growth (Chernin et al., 2011; Garbeva et al., 2014; Audrain et al., 2015; Cordovez et al., 2015; Schmidt et al., 2015), or by affecting gene expression, motility, biofilm formation, and antibiotic resistance of competing bacterial species (Kim et al., 2013; Audrain et al., 2015; Schmidt et al., 2015). Here, we show that VOCs production by plant growth-promoting *Pf.*SBW25 is regulated by the GacS sensor kinase. Our results show that a mutation in the *gacS* gene of *Pf.*SBW25 regulates, independently from growth medium or incubation time, the production of acyclic alkenes and in particular 3-nonene, 1-undecene, and 4-undecyne. These VOCs are produced by several *Pseudomonas* species and have been reported to have growth promoting-effects on *Arabidopsis thaliana* (Blom et al., 2011) and tobacco (*Nicotiana tabacum* cv. Xanthi-nc; (Park et al., 2015). For 1-undecene, Rui et al. (2014) recently identified the biosynthetic gene *undA* in *P. aeruginosa* PA14. Heterologous expression of *undA* homologs of *Pseudomonas* in *E.coli* conferred heterologous expression and production of 1-undecene (Rui et al., 2014). Our previous work showed that a mutation in the Gac-system of *Pf.*SBW25 caused major transcriptomic changes (Cheng et al., 2013). Revisiting these microarray data showed that the *undA* ortholog PFLU4307 in *Pf.*SBW25 was 9.7-fold down-regulated in the *gacS*-mutant. We did not find a so-called Gac-Box (Lapouge et al., 2008; Song et al., 2015) upstream of the *undA* gene in *Pf*.SBW25 (data not shown), suggesting that 1-undecene is not regulated by the Gac-system via small RNAs. The production of VOCs by *Pf.*SBW25 was also affected by the growth medium and growth stage (incubation time), confirming, and extending earlier observations made for VOCs produced by other bacterial and fungal genera (Blom et al., 2011; Schmidt et al., 2016).

Volatile organic compounds differentially produced by *Pf.*SBW25 and the Gac-mutant had no apparent differential effects on growth promotion and ISR in *Arabidopsis*. This observation suggests that the observed phenotypic effects in *Arabidopsis* are caused by VOCs not regulated by the sensor kinase GacS. Another possible explanation may be that other VOCs compensate for the growth-promoting or ISR-inducing VOCs regulated by GacS. Interestingly, tobacco plants did respond differentially to VOCs produced by *Pf.*SBW25 and the Gac-mutant. While VOCs produced by *Pf.*SBW25 repressed growth of the primary root of tobacco seedlings, VOCs from the Gac-mutant promoted tobacco growth relative to the untreated control. Visually, there was a clear difference between the biomass of the roots in the control and the root biomass of tobacco plants exposed to VOCs from *Pf.*SBW25. However, this visual difference was not supported by quantitative measurement, a discrepancy that may be due to differences in other root parameters such as root diameter. The phenotypic effects on root growth also suggests that some of the Gac-regulated VOCs may be toxic to the growth of tobacco seedlings or that the Gac-mutation enhances the production of other VOCs that trigger an increase in root growth and plant biomass. VOCs-mediated effects on plant growth are dose-dependent and can range from deleterious to beneficial effects (Blom et al., 2011; Schmidt et al., 2016). For example, indole has been shown to promote plant growth at low concentrations but to kill plants at high concentrations (Blom et al., 2011). The same was observed for sulfur-containing compounds such as dimethyl disulfide (Kai et al., 2010; Meldau et al., 2013). Therefore, the growth repression of tobacco seedlings by wild-type *Pf.*SBW25 can be due to Gac-regulated VOCs and/or due to specific VOCs that are emitted by *Pf.*SBW25 at higher concentrations than by the Gac-mutant. *Pf.*SBW25 does not produce HCN unlike several other *Pseudomonas* strains, which excludes effects of this VOC on plant growth observed in this study. The Gac-regulated VOCs that qualify for potential adverse effects on tobacco seedling growth include compounds putatively identified as 3-nonene (RI: 891), *n*-decene (RI: 992) 1-undecyne (RI: 1080), 4-undecyne (RI: 1085), 1-undecene (RI: 1089) and, *cis*-1,2-cyclohexanedimethanol (RI: 1280). The absence of these plant-growth-inhibitory VOCs in the Gac-mutant may explain in part the growth promotion observed for the Gac-mutant as compared to the *Pf.*SBW25-treated plants. Plant bioassays with these VOCs, applied alone and in combination in a dose-dependent manner, should be performed to pinpoint those VOCs with toxic or growth-promoting effects on tobacco seedlings. However, this chemical approach has, at this stage, several limitations that may lead to incomplete and inconclusive results. These limitations are: (i) there is a lack of pure references for several of the Gac-regulated VOCs, (ii) a wide range of concentrations and also a large number of combinations of multiple VOCs (at different concentrations) need to be tested, (iii) there is a lack of knowledge on the timing and duration of exposure of the plant seedlings to specific VOCs or combinations of VOCs, and (iv) there are technical limitations to determine if the concentrations used represent biologically relevant concentrations produced by the bacterium *in situ*.

The observed plant-species-specific responses to bacterial VOCs may also be due to differences in VOCs-receptors or down-stream signal transduction pathways between the two plant species tested. To our knowledge, ethylene receptors are the only type of putative receptors reported so far in VOCs perception (Ryu et al., 2003; Bailly and Weisskopf, 2012). Loss of the positive regulator of the ethylene pathway EIN2 led to different VOCs-mediated growth responses in *Arabidopsis* by *Bacillus* strains IN937a and GB03 (Ryu et al., 2003). *Bacillus* VOCs failed to promote growth of *ein2 Arabidopsis* mutants suggesting that the ethylene pathway is involved in the response to bacterial VOCs (reviewed by Bailly and Weisskopf, 2012). Whether similar or other genes and pathways affect the differential responses of the two plant species to VOCs produced by *Pf.*SBW25 will be subject of future analyses.

Next to plant growth promotion, rhizosphere acidification was observed for *Arabidopsis* but not for tobacco seedlings. Farag et al. (2013) showed that *Bacillus* strain GB03 is able to induce rhizosphere acidification via VOCs. The possible mechanisms proposed include elevated proton exudation from roots and direct acidification of the environment by the bacterial VOCs themselves (Farag et al., 2013). VOCs-triggered acidification was also reported to be associated with the enhancement of iron assimilation and photosynthetic efficiency (Farag et al., 2013). Rhizosphere acidification is an efficient way to enhance iron uptake and could explain the enhanced greening of leaves of the VOC-exposed *Arabidopsis* seedlings observed in our study.

## Conclusion

In this study, demonstrated the involvement of the GacS sensor kinase in the regulation of VOCs production in *Pf.*SBW25, which in turn affects plant growth in a species-dependent manner. The GacS significantly affects alkene production in *Pf.*SBW25 independent of the cultivation medium and growth stage. Future studies will focus on the role of these and other VOCs of *Pf.*SBW25 in the differential growth responses observed for *Arabidopsis* and tobacco.

## Author Contributions

XC designed and performed the experiments and drafted the manuscript. VC, DE, and XC analyzed the GC-MS data. MV supervised the work and assisted the writing. JR supervised the work and assisted with the experimental design and writing. All authors revised the manuscript and approved submission.

## Conflict of Interest Statement

The authors declare that the research was conducted in the absence of any commercial or financial relationships that could be construed as a potential conflict of interest.

## Footnotes

1 http://www.nist.gov
